# A Controversial Role for IL-12 in Immune Response and Bone Resorption at Apical Periodontal Sites

**DOI:** 10.1155/2010/327417

**Published:** 2011-02-16

**Authors:** Celso Martins Queiroz-Junior, Marcelo José Barbosa Silva, Jôice Dias Corrêa, Mila Fernandes Moreira Madeira, Thiago Pompermaier Garlet, Gustavo Pompermaier Garlet, Fernando Queiroz Cunha, Mauro Martins Teixeira, Tarcília Aparecida da Silva

**Affiliations:** ^1^Department of Oral Surgery and Pathology, School of Dentistry, Federal University of Minas Gerais, Belo Horizonte, MG, Brazil; ^2^Laboratory of Immunopharmacology, Department of Biochemistry and Immunology, Institute of Biological Sciences, Federal University of Minas Gerais, Belo Horizonte, MG, Brazil; ^3^Department of Microbiology, Institute of Biological Sciences, Federal University of Minas Gerais, Belo Horizonte, MG, Brazil; ^4^Department of Pharmacology, School of Medicine of Ribeirão Preto, University of São Paulo, Ribeirão Preto, SP, Brazil; ^5^Department of Biological Sciences, School of Dentistry of Bauru, University of São Paulo, Bauru, SP, Brazil; ^6^Departamento de Clínica, Patologia e Cirurgia Odontológicas, Faculdade de Odontologia, Universidade Federal de Minas Gerais, Avnida Presidente Antônio Carlos 6627, 31.270-901 Belo Horizonte, MG, Brazil

## Abstract

Periapical lesions are inflammatory conditions of tooth periapical tissues, triggered by dental pulp infection and characterized by exudation of immune cells to the affected tissues and production of inflammatory mediators such as cytokines. The inflammatory periapical reaction is mainly driven by Th1, Th2, and Th17 responses, and such polarization may modulate progression of the disease and expression of bone proresorptive cytokines. IL-12 is a potent inducer of IFN-**γ** production, which stimulates Th1 effector cells. Many evidences have shown a positive correlation between the bone resorptive cytokine IL-1**β** and the production of IL-12 and IFN-**γ**. Furthermore, IL-12 may have a potential role in the release of bone resorptive mediators and blockade of Th2 cytokines, affecting the progression of periapical bone loss. Nevertheless, IL-12 and IFN-**γ** have also been described as suppressors of osteoclast differentiation and activation, favoring bone maintenance. This paper focuses on the controversial roles of IL-12 in periapical lesions.

## 1. Introduction

Interleukin 12 (IL-12) is an important regulatory cytokine that has a pivotal function in the initiation and regulation of cellular immune responses. It can regulate the differentiation of naive T cells, which are crucial in determining resistance and the type of response that will be elicited against a particular pathogen [1]. IL-12 is mainly produced by macrophages, monocytes, dendritic and B cells in response to bacterial products and intracellular parasites. It is also primarily responsible for the subsequent production of interferon-gamma (IFN-*γ*) and tumor necrosis factor-alpha (TNF-*α*) from NK cells and T helper cells. IL-12-induced IFN-*γ* secretion enhances phagocytosis, production of nitric oxide (NO), and oxidative burst, resulting in increased destruction of pathogens [2]. IL-12 also has been well characterized as a suppressor of Th2 cytokines, such as IL-4 and IL-10 [3]. For its roles in immune responses, IL-12 has been implicated in the pathogenesis of several diseases, including inflammatory diseases such as rheumatoid arthritis [4], psoriasis [5], and Crohn's disease [6] and oral conditions such as periodontitis [7]. The aim of this paper is to discuss the mechanisms associated to the IL-12-related immune response in tooth periapical lesions. 

## 2. Periapical Lesions: Concepts and Nomenclature

Dental pulp is protected from microorganisms of the oral cavity by enamel and dentin. The exposure of dental pulp to microorganisms and their products, as a consequence of dental caries, fractures, or operative procedures, triggers a local inflammatory response. The progression of such infection and inflammation results in necrosis of the pulp and consequent involvement of periapical tissues, generating a periapical lesion [8, 9]. In periapical lesions, an initial short acute inflammatory response of varied intensity is accompanied by pain, tooth elevation, and tenderness to percussion. Tissue changes are characterized by hyperemia and neutrophil recruitment, usually limited to the periodontal ligament. With the continuous presence of irritants at periapex, the acute response shifts to the formation of a granulomatous tissue with chronic inflammatory cells and fibroblasts: the apical granuloma [8, 9]. Such condition is asymptomatic and accompanied by a radiolucent area formation as result of periapical bone resorption. A granuloma can remain latent or be converted to inflammatory cysts by poorly understood mechanisms. Cysts are diagnosed as presenting fully developed cavities lined by stratified squamous epithelium with variable thickness and a fibrous capsule [8].

These pathological changes in periapical tissues are the clinical consequence of the host defensive reaction against bacterial products that egress through apical foramen from infected dental pulp [8, 10, 11]. This response is characterized by the persistent migration of polymorphonuclear leukocytes, monocytes, lymphocytes, plasma, and mast cells to the infected sites, and it largely prevents microbial invasion into the periapical tissues [8, 12, 13]. Such immunological response seems to be similar to other reactions to bacterial infections in the body, except for the resorption of the periapical bone [14]. In this regard, although the commitment of immune cells and the consequent production of inflammatory mediators protect the host from pathogen invasion, it may paradoxically account for much of the periapical bone resorption [8, 15].

## 3. Involvement of T Cells in Periapical Lesions

Periapical lesions are marked by the expression of cell surface adhesion molecules, production of chemotactic factors [16, 17], and release of cytokines, including the bone proresorptive interleukin (IL)-1, IL-6, and tumor necrosis factor (TNF)-*α* [14, 18–22]. Such response is primarily regulated by a network of other T-cell-derived cytokines, including members of the IL-12 family [18, 23, 24].

T-helper (Th) cells are the primary cell type responsible for regulating cytokine-mediated immune responses, and the differentiation of naïve CD4^+^ Th cells into effectors T cells is critical [25]. CD4^+^ effector T cells can be divided into distinct lineages such as Th1, Th2, Th17, and regulatory (Treg) cells. In this context, members of the IL-12 cytokine family have abilities to help differentiation and/or maintenance of Th1 cells, and their production is one of the key events in the differentiation of T cell populations. IL-12 and its family members, IL-23 and IL-27, also act as cofactors to enhance T-cell proliferation [3, 26–28]. IL-12 is a potent inducer of interferon (IFN)-*γ* production, which stimulates Th1 effector cells. Chronologically differential roles of IL-12, IL-23, and IL-27 have been proposed for Th1 differentiation: first, IL-27 commits naïve CD4^+^ T cells to differentiate into Th1 cells by activating IL-12 receptor 2, and then IL-12 acts on committed effector Th1 cells stimulating IFN-*γ* production, which is followed by IL-23-mediated proliferation of memory Th1 cells [29]. Also, IL-23 produced by dendritic cells and naïve CD4^+^ T cells induces the differentiation of Th17 cells. IL-23 production is mediated by the recognition of specific structural motifs of various pathogens by Toll-like receptors (TLRs) 2 and 4. The TLR2 and TLR4 agonists (peptidoglycan and LPS, resp.) readily induce IL-12/23p40 expression and, consequently, IL-23 production, favoring Th17 differentiation [30, 31]. Then, Th17-differentiated cells are able to release IL-17, also inducing IFN-*γ* production [26, 32]. 

A common feature of periapical lesions, independent of their underlying cause, is the persistent exudation of a large number of immunocompetent cells, with T cells being predominant cellular components in human periapical pathologies [33–35]. It is well known that the inflammatory periapical reaction is driven by Th1, Th2, Th17, and Treg responses, and such polarization may modulate the expression of bone proresorptive cytokines [18, 32, 36]. Although some studies have supported T cells having a minor role in the pathogenesis of periradicular lesions [37, 38], large amount of evidence indicates that the progression of such disease, with bone resorption, requires Th1 and Th17 cells, while Th2 and Treg cells are related to the chronicity of inflammation [32, 36, 39].

Indeed, periapical lesions do not progress over a long period in congenitally *nu/nu* T cell-deficient mice, which lack a source of T cells. In these animals, the progression of experimentally induced periapical lesions ceased after 6 weeks of infection and was characterized by numerous fibroblasts instead of inflammatory cells in the affected tissues 8 weeks after pulp exposure [40].

Consistent with these findings, some studies demonstrated that Th1 immune response is important for all stages of periapical lesion progression, while Th2 immune modulatory response might be related to asymptomatic and chronic lesions [32, 36, 39]. Along with Th1 and Th2 responses, some recent evidence also implicates IL-17 as an important cytokine responsible for the progression of apical periodontal lesions [32, 41].

Somehow, the development and progression of periapical lesions is dependent on T cells and, consequently, on the mediators released by them.

## 4. IL-12-Related Immune Responses in Periapical Lesions

The expression of most of Th1 cytokines, including IL-2, IL-12, and IFN-**γ**, has been shown to be increased in periapical lesions after experimental pulp exposure. Although the cell source of IL-12 in periapical tissues has not been defined yet, it is probable that macrophages and dendritic cells are responsible for the secretion of such cytokine [42] and that Th1 cells may respond to macrophage-derived IL-12 releasing IFN-*γ* and IL-2 [43]. On the other hand, the expression of Th2-type cytokines was similarly increased in periapical tissues, but had declined at the latest time-point, suggesting a possible inhibition by Th1-type mediators [18]. In fact, IL-12 also has been well characterized as a suppressor of Th2 cytokines, such as IL-4 [3]. This immunological interaction could support the destruction of periapical tissues. In this regard, significant correlations were observed between levels of IL-1**α** and Th1-derived proinflammatory mediators IL-2, IL-12, TNF-**α**, and IFN-*γ* [18]. Nevertheless, there was a lack of correlation between IL-1**α** and Th2-type anti-inflammatory mediators, including IL-4, IL-6, and IL-10 [18]. These data point toward a potential role of IL-12 in the release of proinflammatory mediators and blockade of Th2 cytokines, which could favor the progression of infection-induced periapical lesions. Besides this Th1/Th2 paradigm, Th17 cells were recently described as having a role in the IL-12-related immune response in apical lesions. Xiong et al. [44] reported that the number of cells expressing IL-17 increased along the postoperative time in experimentally induced periapical lesion in rats, and Marçal et al. [41] showed that the frequency of IL-17 positive cells was significantly higher in radicular cysts and granulomas than in healthy human tissues. IL-17 secreted by Th17 cells can induce IFN-*γ* production by differentiated CD4^+^ T cells exacerbating the inflammatory process [32]. In addition, IL-17 is able to augment the expression of the neutrophil chemokines CXCL1 and CXCL5, as demonstrated in experiments using IL-17R^−/−^ mice, favoring the host response against periodontopathogens [45]. Despite this new insight of Th17 response in the understanding of the IL-12-related immune pathway in periapical lesions, not much of it is known yet, and the current broad knowledge still focuses on Th1 and Th2 responses.

Previous studies from our group pointed towards a predominance of distinct Th cell types in human periapical conditions being Th1 markers associated with granuloma, while Th2 mediators augmented in cysts. However both lesions exhibited similar expression of IL-4 and IFN-*γ* [16, 36]. Conversely, other investigation has found a regulatory environment in granulomas, with high transforming growth factor (TGF)-*β* and low proinflammatory cytokine levels. In contrast, periapical cysts were characterized by a Th1 and Th2 response, with increased IFN-*γ*, TNF-*α*, and IL-4 levels which were correlated to clinical evidence of swelling and tenderness to percussion [46]. Other studies suggest that Th1 response is predominant in apical granulation tissues, while Th2 response is dominant in human periapical regenerating lesions [21, 47]. On the other hand, experimental models suggest a hierarchy of Th2 cytokines in the immunomodulation of apical periodontitis, given that the absence of Th1-type cytokines (IFN-*γ* and IL-12) does not interfere with the lesion development [23, 24], whereas the deficiency of Th2 cytokines, IL-6 [20] and IL-10 [23, 48], increases the extension of apical lesions. 

In order to determine the individual function of the Th1-driven cytokines IL-12 and IFN-*γ* in the pathogenesis of periapical bone destruction, Sasaki et al. [24] used a well-established model of periapical lesion in appropriate knockout mice. Periapical lesion was induced by inoculation of a bacterial load into the root canal system of mice's 1st molars, and their results indicated that IL-12^−/−^ and IFN-*γ* ^−/−^ mice had similar bone resorption *in vivo*, compared to wild-type mice. Infusion of recombinant IL-12 in infected conventional animals (in order to detect whether a high concentration of the cytokine could induce any alteration in bone resorption) also did not alter periapical bone loss. Herein, an *in vitro* study was performed, and they demonstrated that recombinant IL-12 and IFN-*γ* failed to modulate macrophage IL-1*α* production [24]. Thus, at least individually, IL-12 and IFN-*γ* did not seem to have a significant effect on the pathogenesis of periapical lesions *in vivo*. It is important to consider that the individual effect of leukocytes subsets and cytokines is usually investigated in highly controlled systems, while *in vivo* the putative function of cytokines must be estimated in the view of a complex milieu, with presence of several other cytokines, which can modulate or be modulated by them until the determination of a clinical outcome.

Nevertheless, in sharp contrast, another research group found divergent results in IFN-*γ* ^−/−^ mice, essentially using a similar experimental model [23]. IFN-*γ* ^−/−^ animals presented an increased periapical bone loss in relation to wild-type animals, but a reduced neutrophil number associated to a relative increase in the number of mononuclear cells at the periapical region. Bacterial quantification inside the root canal system and the number of osteoclasts in the periapical bone were similar to wild-type mice. These results suggest a role for IFN-*γ* as a suppressor of periapical lesion bone destruction [23]. Some methodological differences could explain these divergent results, such as the quantitative method used to analyze bone loss (microcomputed tomography versus histological analysis) and the different bacterial strains used to induce pulp infection, which could be responsible for distinct patterns of bone resorption [23, 24]. In fact, the presence of specific pathogens is able to interfere with cytokine milieu, turning *in vivo* models scenario with multiple bacterial species even more complex to be evaluated, but possibly more close to mimic human lesions. Despite the controversy, both studies suggested a possible functional redundancy in pro- and anti-inflammatory pathways related to periapical lesions. Although the IL-12-IFN-*γ* pathway is immunologically described as a predominantly Th1 proinflammatory and bone-destructive-inducing system, it has also been described as a suppressor of osteoclast differentiation and activation [49, 50].

The alteration in the balance of these opposing processes—proinflammatory signals to prevent infection versus osteoclast inhibitory activity—could explain the apparent lack of effect of IL-12 in the control of pulp infection and periapical lesion development.

## 5. Effects of IL-12 on Periapical Bone Resorption

Periapical bone resorption is caused by the imbalance between osteoblast and osteoclast activity. Bone formation can be driven by factors such as bone morphogenetic proteins, cytokines, and growth factors, which induce the differentiation of precursor cells into osteoblast phenotype [51–53]. Once differentiated, the osteoblasts produce several proteins which will compose newly formed bone [54] and then undergo differentiation under osteocyte phenotype [55]. While no data is available in the literature concerning the expression of bone formation markers specifically in periapical lesions, recent studies demonstrate that inflammatory cytokines (described to be upregulated by Th1 responses) interfere in coupled bone formation (the process of equivalent bone formation of the amount of bone resorbed, which takes place under homeostatic conditions) [56, 57] suggesting that this phenomenon may also account for bone loss in periapical lesions. On the other hand, differentiation and activation of osteoclasts, and consequent bone resorption, is driven by RANK (receptor activator of nuclear factor-*κ*B), its ligand RANKL, and its soluble counterpart OPG (osteoprotegerin) [58]. RANKL binding to the receptor RANK, present on the surface of preosteoclasts, drives their maturation and activation, while OPG acts as a decoy receptor and inhibits RANK-RANKL engagement [58]. Interestingly, periapical granulomas present with heterogeneous patterns of RANKL and OPG expression, ranging from samples with RANKL/OPG ratio similar to that seen in sites with absent bone resorption to patterns indicative of active bone resorption [59]. Such imbalance is triggered by the main cytokines produced during the acute phase of periapical lesion: RANKL, IL-1*α*, IL-1*β*, and TNF-*α* [10, 11, 19, 60–63]. Many evidences have shown a positive correlation between the bone resorptive cytokine IL-1*β* and the production of IL-2, IL-12, TNF-*α*, and IFN-*γ* [18, 19]. Most of them are Th1 cytokines, and they are produced during the acute phase of periapical lesions. On the other hand, the chronic phase of disease is characterized by the production of Th2 cytokines (IL-4, IL-6, IL-10, and IL-13) that reduce the bone resorption activity [7]. Such affirmation was demonstrated by experiments using IL-10^−/−^ mice. The absence of IL-10 leads to the development of larger periapical lesion compared to wild-type mice. Besides, the levels of IL-1*β* and IL-12 were remarkably higher compared to wild-type animals, indicating that IL-10 acts as a suppressor of IL-1*β* and IL-12 overproduction [7, 48]. Other Th2 cytokines like IL-4 and IL-13 can also inhibit bone resorption by reducing the production of Th1 cytokines [19]. Paradoxically, gene knockouts of the prototype Th1 mediator interferon (IFN)-*γ* or IFN-*γ*-inducing cytokines IL-12 and IL-18 have no significant effect on periapical bone destruction, suggesting either a lack of regulatory activity or functional redundancy in proinflammatory pathways [24].

**Figure 1:**
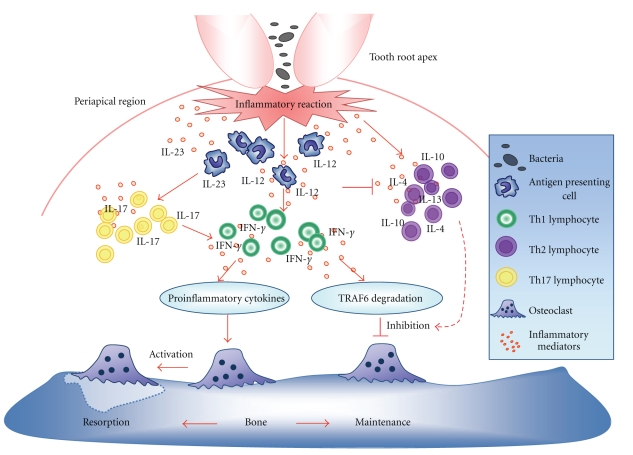
Schematic drawing showing the controversial role of IL-12 on bone resorption at apical periodontal sites. The progression of dental pulp infection triggers an inflammatory response in periapical tissues. Inflammatory cells, such as macrophages, are recruited for that region and release proinflammatory mediators. One of those mediators is IL-12, which induces Th1 cells to produce IFN-*γ*. The IL-12-IFN-*γ* pathway can induce bone resorption by production of proinflammatory cytokines, such as TNF-*α* and IL-1*β*, which leads to the activation of osteoclasts. In contrast, this pathway is also involved in the degradation of the RANK adapter protein, TRAF6, which reduces RANKL-induced osteoclast differentiation. In this context, dendritic cells and naïve CD4^+^ cells also produce the IL-12 family member cytokine IL-23, which induces the differentiation of Th17 cells. These cells release IL-17, enhancing the IFN-*γ* production. In favor of this proinflammatory environment IL-12 also may blockade Th2 cytokines, stimulating the progression of infection-induced periapical lesions. These opposing mechanisms may explain the discrepant findings regarding IL-12 and IFN-*γ* in the pathogenesis of periapical lesions. IFN-*γ*: interferon-*γ*; RANK: receptor activator of nuclear factor *κ*B; TRAF6: tumor necrosis factor receptor-associated factor 6.

It can be considered that IL-12 has dichotomic effects related to osteoclastogenesis as illustrated in Figure 1. One of those is the induction of osteoclast differentiation by enhancing the production of IL-1*β* and Th1 cytokines and provoking destructive osteolysis around root apex during the development of periapical lesion [19]. However, *in vitro* studies demonstrated that IL-12 indirectly reduces RANKL-induced osteoclast differentiation alone or in synergy with IL-18 [64]. Moreover, it seems that T cells play an important role in the osteoclastogenesis inhibition by IL-12 since their absence in these cultures ablated the effects of IL-12 treatment [64]. Other group showed that IFN-*γ* also suppresses RANKL-induced osteoclast differentiation and mature osteoclast function by enhancing the IFN-*γ*-induced degradation of the RANK adapter protein, TRAF6 (tumor necrosis factor receptor-associated factor 6), which results in strong inhibition of the RANKL-induced activation of the transcription factors NF-*κ*B and JNK [50]. Interestingly, genetic ablation of chemokine receptor CCR5, characteristically expressed by Th1 polarized lymphocytes, results in formation of larger periapical lesions [23], reinforcing the potential protective role of IFN-*γ* in bone lytic lesions. Therefore, we can wonder that the inhibitory effects of IL-12 in the osteoclast differentiation are at least in part dependent on IFN-*γ* production by T-cells. However, the proinflammatory effect of IFN-*γ* demonstrated *in vivo*, which results in the upregulation of the levels of TNF-*α* and IL-1*β* (and consequently RANKL), seems to overcome the direct antiosteoclastogenic effect described *in vitro* [65, 66]. In addition, IFN-*γ* also stimulates osteoclast formation and bone loss *in vivo* via antigen-driven T cell activation or through the chemoattraction of RANKL^+^ cells [65–67]. In accordance with the potential destructive role of IL-12-driven and IFN-*γ*-mediated Th1 responses in periapical lesions, unpublished data from our research group support this hypothesis. When periapical granulomas were categorized into active or inactive based on the RANKL/OPG expression pattern (as previously cited) [59], our results demonstrate that the expression of both IL-12 and IFN-*γ* is significantly higher in active lesions, while Th2- and Th22-type cytokines prevail in inactive lesions.

Moreover, although no data is available directly regarding periapical lesions, the recently described IL-23/IL-17 axis also might account for the divergent effects of IL-12 on bone resorption. IL-23 is a heterodimeric cytokine consisting of an IL-12p40 subunit coupled with IL-23-specific p19 subunit. It shares with IL-12 the IL-12R*β* heterodimer in their receptors, activating many of the same signaling molecules and transcription pathways [68, 69]. In a model of arthritis, Th17 cells stimulated by IL-23 promote osteoclastogenesis through production of IL-17, which in turn induces mesenchymal cells to release RANKL [69, 70]. IL-23 can also stimulate osteoclast formation by direct action on myeloid precursors (inducing RANK expression) and indirectly on osteoblasts (upregulating RANKL expression) [69]. A recent study showed that IL-23 dose-dependently upregulates RANK expression in murine bone marrow macrophages and RAW264.7 cells promoting osteoclastic differentiation in an IL-17-independent pathway. The results also showed that IL-23 synergizes with RANKL to drive osteoclast formation but IL-23 by itself is not able to induce osteoclastogenesis [71]. Nevertheless, like IL-12, IL-23 can act synergistically with IL-18 to block osteoclastogenesis in a CD4^+^ T cell-dependent manner *in vitro*. Interestingly, IL-23 does not seem to mediate IL-12 action, although IL-12 can induce its expression [72]. Also, osteoclastogenesis from bone marrow cells induced by soluble RANKL was partially inhibited by IL-23, with reduced multinucleated cell numbers, but this interleukin did not affect the proliferation of osteoclast progenitor cells [73]. These findings point to a yet divergent scenario of this cytokine in conditions involving bone resorption. Therefore, an approach comprising the evaluation of multiple instead of individual cytokines would provide a broader basis to define the cytokines role in periapical lesions outcome, since it takes in account the overall balance of cytokines with opposing or similar functions. Also, the determination of the putative disease activity by means of RANKL/OPG pattern overcomes the absence of definitive clinical data concerning the actual disease activity (i.e., active bone resorption), which certainly contribute to the conflicting results regarding the role of IL-12 (and also the other several cytokines involved) in periapical lesions development. Finally, the growing application of experimental models using genetically modified mice strains allows the cause-and-effect relationships, providing important contributions to the study of the immunopathogenesis of periapical pathologies [63, 74, 75].

## 6. Concluding Remarks

The development and progression of periapical lesions is markedly dependent on the inflammatory reaction triggered by pulp infection. IL-12 is related to the differentiation of Th1 cells, and there is evidence that Th cells are directly involved in the progression of periapical lesions and bone resorption. Th1 cells respond to macrophage-derived IL-12 releasing IFN-*γ* and suppressing Th2 cytokines, favoring the infection-induced bone loss. Thus, the IL-12-IFN-*γ* pathway may contribute to the progress of periapical lesions due to its proinflammatory actions. Furthermore, Th17 cells differentiated upon IL-23 production also seem to stimulate proinflammatory tooth periapical reaction through IL-17-induced IFN-*γ* release. Some studies showed that, individually, there is no effect of IL-12 and IFN-*γ* in the pathogenesis of periapical lesions. The proinflammatory role of IL-12 in these lesions seems to be counterbalanced by its inhibitory effects in the osteoclast differentiation, which, at least in part, is also dependent on IFN-*γ* production. In conclusion, IL-12 seems to have a dual role in the pathogenesis of periapical lesions.
